# The phase diagram and stability of trapped *D*-dimensional spin-orbit coupled Bose-Einstein condensate

**DOI:** 10.1038/s41598-017-15900-w

**Published:** 2017-11-15

**Authors:** Zi-Fa Yu, Ju-Kui Xue

**Affiliations:** College of Physics and Electronic Engineering, Northwest Normal University, Lanzhou, 730070 China

## Abstract

By variational analysis and direct numerical simulation, we study the phase transition and stability of a trapped *D*-dimensional Bose-Einstein condensate with spin-orbit coupling. The complete phase and stability diagrams of the system are presented in full parameter space, while the collapse dynamics induced by the mean-filed attraction and the mechanism for stabilizing the collapse by spin-orbit coupling are illustrated explicitly. Particularly, a full and deep understanding of the dependence of phase transition and stability mechanism on geometric dimensionality and external trap potential is revealed. It is shown that the spin-orbit coupling can modify the dispersion relations, which can balance the mean-filed attractive interaction and result in a spin polarized or overlapped state to stabilize the collapse, then changes the collapsing threshold dependent on the geometric dimensionality and external trap potential. Moreover, from 2D to 3D system, the mean-field attraction for inducing the collapse is reduced and the collapse speed is enhanced, namely, the collapse can be more easily stabilized in 2D system. That is, the collapse can be manipulated by adjusting the spin-orbit coupling, Raman coupling, geometric dimensionality and the external trap potential, which can provide a possible way for elaborating the collapse dynamics experimentally.

## Introduction

In the past decades, as the development of the laser cooling technique, ultracold neutral atoms provide an ideal platform for quantum simulations due to its purity, highly controllability and effortless observability^1^. Particularly, through the synthetic gauge field, the NIST Group has successively realized uniform vector potential^2^, synthetic magnetic fields^3^, electric fields^4^, and spin-orbit coupling (SOC)^5^ in a ^87^Rb Bose-Einstein condensate (BEC) during 2009 to 2011, which makes it possible to simulate the properties of the charged particle in an electromagnetic field by using a neutral atom. In the recent experimental^5–8^ and theoretical^9–16^ research of synthetic SOC BEC, some new quantum phases possessing distinct magnetic features have been exhibited, such as non-magnetic zero momentum phase, magnetic plane wave phase and non-magnetic stripe phase. However, the stability plays an important role in the realization of BEC experimentally. Without SOC, the stability of the BEC closely depends on the interatomic interactions, the characteristic scale, and the geometric dimensionality. It mainly demonstrates as the strongly repulsive diffusion and the strongly attractive collapse^17–22^. In a trap potential, this stability prevailingly presents as the collapse produced by quantum pressure due to attractive interaction. In addition, the collapse is also related to the research of the fundamental physics, such as all kinds of nonlinear systems^23^, plasma instability^24^, polaron formation^25^, and cold dark matter halos^26^. Recently, the collapse dynamics of BEC has been attracted more and more attentions^27–30^. There have been already several proposals to stop the collapse of attractive condensate, including by making the interaction strength time-dependent, and by adding fermions to the system, etc. Moreover, SOC can stabilize the collapsed BEC by means of modifying the dispersion relations^31–38^. It is shown that the stabilizing mechanism of SOC strongly depends on the geometric dimensionality and the external trap potential. In two-dimensional (2D) free space^35^, the conventional BEC exists the Galilean invariance, which can nevertheless be broken by the SOC term, thus the BEC can be stabilized and a stable solitonlike structure is formed, while in 2D external trap potential^37^, an effective repulsive atomic interaction produced by the SOC and the Raman coupling (RC) can neutralize the mean-field attractive interaction, stabilize the system against collapse, change the stability criteria, and generate various phases. In 3D free space^38^, the SOC-induced modification of the dispersion relations of the BEC can neutralize the attraction, creating metastable solitons and forming the semivortices or mixed mode structures. However, for a trapped 3D binary BEC with SOC (a more realistic case in experiment), the stability of various phases has never been obtained, and the stability mechanism is still not clear. Particularly, a full and deep understanding of the dependence of phase transition and stability mechanism on geometric dimensionality and external trap potential is still missing.

**Figure 1:**
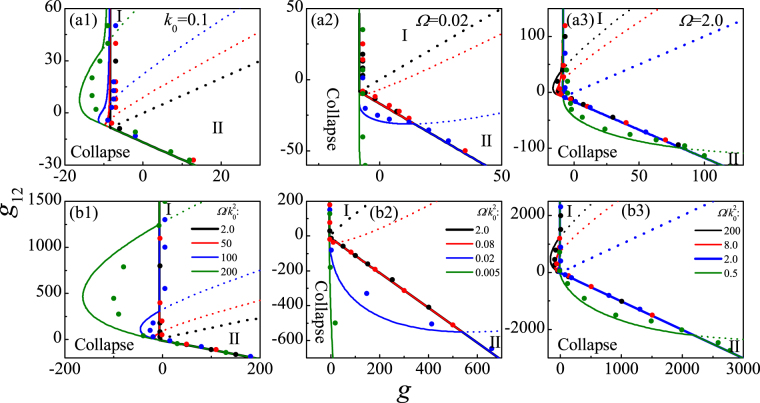
The stability and phase diagram in *g*–*g*_12_ plane for *D* = 3 (**a1**–**a3**) and *D* = 2 (**b1**–**b3**). The solid and short dashed line, respectively, refers to stability and phase boundary by means of the variational methods. The dots represent the results of direct numerical simulation of Eq. (1). The solid line divides the interaction plane into two, one is collapse region the other is stable region, while the stable region is divided into phase I (zero momentum phase) and phase II (plane wave phase) by the short dashed line.

The purpose of this work is to study the distinct phases and their stability, and the stability mechanism of a trapped *D*-dimensional binary BEC with SOC. The collapsing threshold, the phase and stability diagrams and the collapse dynamics are presented by variational methods and confirmed by the direct numerical simulation of Gross-Pitaevskii equation in full parameter space. The main results are summarized in Fig. 1, where the complete stability and phase diagrams are depicted in intra- and inter-species interaction (*g*–*g*_12_) plane for different SOC, RC and geometric dimensionality. For a conventional BEC (i.e., without SOC and RC), the collapse will occurs for the attractive interactions beyond the threshold. However, the strong RC Ω and weak SOC *k*_0_ (i.e., ${\rm{\Omega }}/{k}_{0}^{2} > 2$) can stabilize the collapsed conventional BEC induced by strong intra-species attractive interaction *g*, then result in a stable phase I (i.e., the zero-momentum phase). As the increase of ${\rm{\Omega }}/{k}_{0}^{2}$ (see Fig. 1(a1,b1,a3,b3)), the critical intra-species attractive interaction value for collapse increases, thus the BEC with a stronger intra-species attraction is also stable, while the region of phase I becomes larger and the region of phase II (the plane wave phase) becomes smaller. On the other hand, the collapsed BEC with strong inter-species attractive interaction *g*_12_ can be stabilized by weak RC and strong SOC (i.e., ${\rm{\Omega }}/{k}_{0}^{2} < 2$), which generates a stable phase II. As the decrease of ${\rm{\Omega }}/{k}_{0}^{2}$ (see Fig. 1(a2,b2,a3,b3)), the threshold of collapse shifts down to stronger inter-species attraction, thus the BEC with a stronger inter-species attraction is also stable, while the regions of phase I (phase II) decreases (increases). Furthermore, a full and deep understanding of the dependence of phase transition and stability mechanism on geometric dimensionality and external trap potential is presented explicitly. Compared with the 3D case (Fig. 1(a1,a2,a3)), the regions of stable BEC are expanded, the collapse threshold is enhanced, the collapse speed is reduced, and the collapse can be more easily prevented in 2D external trap potential (Fig. 1(b1,b2,b3)), which can provide a possible way for elaborating the collapse dynamics.

## Results

### Model

Based on the recent experiment related to the realization of BEC with SOC^5–8^, we consider a binary SOC-BEC loaded into a *D*-dimensional harmonic trap potential. According to the mean-field approach, the BEC is governed by the following dimensionless Gross-Pitaevskii (GP) equation^5–7,12–15^:

1 $${\rm{i}}\partial {\boldsymbol{\Psi }}({\bf{r}},\,t)/\partial t=({h}_{0}^{{\rm{SO}}}+G)\,{\boldsymbol{\Psi }}({\bf{r}},t),$$

where **Ψ** = (*ψ*_↑_,*ψ*_↓_)^*T*^ is the normalized spinor wave function so that $\int \,d{\bf{r}}{|{\boldsymbol{\Psi }}|}^{2}=1$, $G={\rm{diag}}({g}_{11}{|{\psi }_{\uparrow }|}^{2}+$ ${g}_{12}{|{\psi }_{\downarrow }|}^{2},{g}_{22}{|{\psi }_{\downarrow }|}^{2}+{g}_{12}{|{\psi }_{\uparrow }|}^{2})$ characterizes the interatomic two-body interaction with the dimensionless interaction constants ${g}_{ij}=\mathrm{2(2}\pi {)}^{(D-\mathrm{1)/2}}N\hslash {a}_{ij}/(m{\omega }_{z}{l}_{z}^{3})$, here, *m*, *N*, and ${l}_{z}=\sqrt{\hslash /(m{\omega }_{z})}$ represents the atomic mass, the number of atom, and characteristic length along *z* direction with trapped frequency *ω*_*z*_, respectively. The positive (negative) *s*-wave scattering lengths *a*_*ij*_ refers to the interatomic repulsion (attraction). The physical variables are rescaled as ${\boldsymbol{\Psi }}\sim {l}_{z}^{D\mathrm{/2}}{\boldsymbol{\Psi }}$, $t\sim {\omega }_{z}^{-1}t$, ${\bf{r}}\sim {l}_{z}{\bf{r}}$. The single-particle Hamiltonian

2 $${h}_{0}^{{\rm{SO}}}=\frac{1}{2}[({p}_{x}-{k}_{0}{\sigma }_{z}{)}^{2}+{p}_{\perp }^{2}]+\frac{{\rm{\Omega }}}{2}{\sigma }_{x}+{V}_{{\rm{ext}}},$$

where ${\rm{\Omega }}=\tilde{{\rm{\Omega }}}/(\hslash {\omega }_{z})$ is the dimensionless RC constant accounting for the transition between the two spin states, ${k}_{0}=\tilde{{k}_{0}}/(\hslash {l}_{z}^{-1})$ is the dimensionless SOC strength fixed by the momentum transfer of the two Raman lasers, the operator ${\bf{p}}=-{\rm{i}}\nabla$ is the canonical momentum, *σ*_*i*_ are the usual 2 × 2 Pauli matrices, while ${V}_{{\rm{ext}}}=\frac{1}{2}{\omega }^{2}{{\bf{r}}}^{2}$ is a harmonic trap with $\omega \sim 1$ for *D* = 3 due to the spherical symmetry and $\omega ={\omega }_{{\parallel }}/{\omega }_{z}\ll 1$ for *D* = 2 due to the pancake-shaped cylindrical symmetry where ${\omega }_{{\parallel }}$ is the transverse trapped frequency. Note that, there is a peculiar property of violating both parity and time-reversal symmetry in Hamiltonian Eq. (2). Here, we have *g*_11_ = *g*_22_ = *g* in the absence of Zeeman splitting. Based on experiment^5,39,40^, for ^87^ *Rb* atoms, corresponding parameters can be widely adjusted, which has already been estimated in the previous work^37^.

From the scale analysis, the amplitude of normalized spinor wave function can be estimated, i.e., $|{\boldsymbol{\Psi }}|\sim {L}^{-D\mathrm{/2}}$, where *L* is the dimensionless characteristic size of BEC. Therefore the dependence of the system’s energy on the characteristic size is obtained as $E(L)\sim {c}_{kin}{L}^{-2}+({c}_{int}^{intra}+{c}_{int}^{inter}){L}^{-D}+{c}_{trap}{L}^{2}-{c}_{soc}{L}^{-1}$, where coefficient *c*_*kin*_, *c*_*trap*_ and *c*_*soc*_ are all positive, while positive (negative) ${c}_{int}^{intra}$ and ${c}_{int}^{inter}$ refers to intra- and inter-species repulsive (attractive) interaction, respectively. It is well known that the external trap potential changes the dispersion relations, which can balance the mean-field repulsive interaction and result in stable BEC instead of diffusion. Similarly, the SOC can also modify the dispersion relations, which suggests a way to neutralize the mean-field attractive interaction to stabilize the collapsed BEC dependent on the external trap potential and the geometric dimensionality. A full and deep understanding of the dependence of phase transition and stability mechanism on the coupling effects of SOC, RC, geometric dimensionality, external trap potential and interatomic interaction is presented explicitly in following by means of accurate variational analysis.

### Variational analysis for phase transition and stability

In order to obtain the stability diagram and the collapse dynamics of trapped *D*-dimensional binary BEC with SOC, while centers around the zero momentum and plane wave states, the following normalized spinor order parameter is convenient and applicable^37^,

3 $${\boldsymbol{\Psi }}({\bf{r}},t)=(\begin{array}{c}{\psi }_{\uparrow }\\ {\psi }_{\downarrow }\end{array})=\frac{{{\rm{e}}}^{-\frac{{{\bf{r}}}^{2}}{2{R}^{2}}+{\rm{i}}{\bf{k}}\cdot {\bf{r}}+\frac{{\rm{i}}\delta }{2}{{\bf{r}}}^{2}}}{\sqrt{2}{(\sqrt{\pi }R)}^{D\mathrm{/2}}}(\begin{array}{c}{{\rm{e}}}^{\frac{{\rm{i}}\varphi }{2}}\sqrt{1+s}\\ -{{\rm{e}}}^{-\frac{{\rm{i}}\varphi }{2}}\sqrt{1-s}\end{array}),$$

with the width of the BEC *R* (*R* > 0), momentum **k** = (*k*_*x*_, *k*_*y*_, *k*_*z*_), the variational rate of radius *δ*, the phase difference between two pseusospin states *ϕ*, and the average spin polarization *s* (−1 ≤ *s* ≤ 1), i.e., 〈*σ*_*z*_〉 = *s*. Upon substitute the spinor wave function into the Lagrangian ${\mathcal L} =\int \,[({\rm{i}}\mathrm{/2})\,({{\boldsymbol{\Psi }}}^{\ast }\dot{{\boldsymbol{\Psi }}}-{\boldsymbol{\Psi }}{\dot{{\boldsymbol{\Psi }}}}^{\ast })-{{\boldsymbol{\Psi }}}^{\ast }({h}_{0}^{{\rm{SO}}}+G){\boldsymbol{\Psi }}]d{\bf{r}}$, one can obtain

4 $$ {\mathcal L} =-\frac{D}{4}{R}^{2}\dot{\delta }-\frac{1}{2}s\dot{\varphi }-E,$$

where

5 $$E=\tfrac{1}{2}{{\bf{k}}}^{2}+\tfrac{1}{2}{k}_{0}^{2}-{k}_{0}{k}_{x}s+\tfrac{D}{4{R}^{2}}+\tfrac{g\mathrm{(1}+{s}^{2})+{g}_{12}\mathrm{(1}-{s}^{2})}{\mathrm{4(2}\pi {)}^{D\mathrm{/2}}{R}^{D}}+\tfrac{D}{4}{\omega }^{2}{R}^{2}+\tfrac{D}{4}{R}^{2}{\delta }^{2}-\tfrac{{\rm{\Omega }}}{2}\sqrt{1-{s}^{2}}\,\cos \,\varphi .$$

Applying the Euler-Lagrangian equations $\partial  {\mathcal L} /\partial {q}_{i}-d(\partial  {\mathcal L} /\partial {\dot{q}}_{i})/dt=0$, where *q*_*i*_ = {**k**, *R*, *δ*, *s*, *ϕ*}, we arrive at

6 $${\bf{k}}=({k}_{0}s,0,\mathrm{0),}$$

7 $$\dot{s}={\rm{\Omega }}\sqrt{1-{s}^{2}}\,\sin \,\varphi ,$$

8 $$\dot{\varphi }=s\,[2{k}_{0}^{2}-\frac{g-{g}_{12}}{{\mathrm{(2}\pi )}^{D\mathrm{/2}}{R}^{D}}-\frac{{\rm{\Omega }}\,\cos \,\varphi }{\sqrt{1-{s}^{2}}}],$$

9 $$\ddot{R}+{\omega }^{2}R=\frac{1}{{R}^{3}}+\frac{g\mathrm{(1}+{s}^{2})+{g}_{12}\mathrm{(1}-{s}^{2})}{\mathrm{2(2}\pi {)}^{D\mathrm{/2}}{R}^{D+1}}.$$

Obviously, in the ground state, the BEC is located at a momentum state (*k*_*m*_, 0, 0), where *k*_*m*_ = *k*_0_ *s*. For the equilibrium state, one has *δ* = 0 and *ϕ* = 0, while *s* and *R* can be determined by the stationary equation of Eqs (8) and (9). The width of the condensate *R* is closely related to SOC and RC, thus the collapse can be manipulated by SOC and RC. The distinct phases of the ground state can be distinguished by the stationary solution of Eqs (7–9), while the breather dynamics of the BEC can be described by the evolution of Eqs (7–9).

On the other hand, in order to acquire a stable equilibrium state, there is not a negative eigenvalue in the corresponding Hessian matrix, i.e., $({\partial }^{2}E/\partial {s}^{2})\,({\partial }^{2}E/\partial {R}^{2})-{({\partial }^{2}E/\partial R\partial s)}^{2} > 0$ and ∂^2^ *E*/∂*R* ^2^ > 0, which results in

10 $$[D(D+\mathrm{2)}{\omega }^{2}+\tfrac{D\mathrm{(2}-D)}{{R}^{4}}]\,[\tfrac{g-{g}_{12}}{{\mathrm{(2}\pi )}^{D\mathrm{/2}}{R}^{D}}+\tfrac{{\rm{\Omega }}}{{\mathrm{(1}-{s}^{2})}^{\mathrm{3/2}}}-2{k}_{0}^{2}]-\tfrac{{D}^{2}{(g-{g}_{12})}^{2}{s}^{2}}{{\mathrm{(2}\pi )}^{D}{R}^{\mathrm{2(}D+\mathrm{1)}}} > 0,$$

11 $$[D(D+\mathrm{2)}{\omega }^{2}+\tfrac{D\mathrm{(2}-D)}{{R}^{4}}] > 0.$$

Hence, the stable equilibrium state can be obtained by the stationary equations of Eqs (8–11). In an external trap potential, the instability only demonstrates as collapse induced by strong interatomic attractive interaction. Thus, the BEC is unstable for *R* → 0, i.e., collapse occurs. However, for a stable ground state, *R* should be finite, where some distinct phases are demonstrated, which depends on *s*.

### Phase and stability diagrams

Here, we focus on distinct phases, the stability boundary and stabilizing mechanism of 3D system, while discuss the difference of phase transition and stability between 2D and 3D system. In 3D system, the ground state can not been obtained analytically and the boundaries of the phase transition and stability can not be depicted analytically, which can only be described numerically by using Eqs (7–11), however, in particular cases, the analytical conditions of phase transition and stability can been acquired.

#### *Case I*

: For *g* = *g*_12_, the condition of phase transition can be obtained by the stationary solution of Eqs (8, 10 and 11). When ${\rm{\Omega }}/{k}_{0}^{2} > 2$, the system enters into a non-polarized zero momentum state (phase I) in the ground state, where **k** = (0, 0, 0) due to *s* = 0. When ${\rm{\Omega }}/{k}_{0}^{2} < 2$, the BEC condenses into a plane wave state with non-zero momentum **k** = (*k*_*m*_, 0, 0) due to $s=\pm \sqrt{1-{{\rm{\Omega }}}^{2}\mathrm{/(4}{k}_{0}^{4})}\ne 0$ in the ground state, where ${k}_{m}=\pm {k}_{0}\sqrt{1-{{\rm{\Omega }}}^{2}\mathrm{/(4}{k}_{0}^{4})}$, which is a polarized plane wave phase (phase II). The phase II breaks the parity symmetry, time-reversal symmetry, and *U*(1) gauge symmetry. Therefore, the condition of phase transition is defined as ${\rm{\Omega }}/{k}_{0}^{2}=2$, which is independent on the geometric dimensionality and external trap potential, meanwhile the SOC (RC) leads to the BEC translating from phase I (II) to phase II (I). Otherwise, in this case, Eq. (10) always stands up if Eq. (11) is satisfied, while Eq. (11) can be written as *R* > ((*D* − 2)*ω* ^−2^/(*D* + 2))^1/4^. By inserting it into the stationary equation of Eq. (9), the stability condition can be obtained as

12 $$g={g}_{12} > -c,$$

where

13 $$c=\mathrm{[4(2}\pi {)}^{D\mathrm{/2}}{(D-\mathrm{2)}}^{(D-\mathrm{2)/4}}]/[(D+{\mathrm{2)}}^{(D+\mathrm{2)/4}}{\omega }^{(D-\mathrm{2)/2}}],$$

i.e., *c* = 2*π* for *D* = 2 and *c* = 8*π*(2*π*)^1/2^/(5^5/4^ *ω* ^1/2^) = 2.68*π* for *D* = 3. Thus, in this case, the phase transition only depends on SOC and RC, while the stability criteria only depends on geometric dimensionality and external trap potential.

#### *Case II*

: For ${\rm{\Omega }}=2{k}_{0}^{2}$ or Ω = *k*_0_ = 0, the phase transition only depends on *g*/*g*_12_ (see Eq. (8)). The BEC is in phase I for *g*_12_ < *g* and in phase II for *g*_12_ > *g*. Thus the intra-species (inter-species) repulsion promotes the system to convert from phase II (I) into phase I (II). Therefore, the phase transition has nothing to do with SOC, RC, geometric dimensionality and external trap potential. In this case (i.e., Ω = *k*_0_ = 0), by using Eqs (8–11), the stability condition can be obtained as

14 $$\begin{array}{lll}\quad \quad g &  >  & -c,\\ g+{g}_{12} &  >  & -2c.\end{array}$$

Thus, the collapse occurs if Eq. (14) is not satisfied, i.e., as *g*_12_ = 0, the collapsing critical intra-species attraction *g*_cri_ = −*c* = −2.68*π* for *D* = 3 and *g*_cri_ = −*c* = −2*π* for *D* = 2^37^. This agrees with the previous theoretical^17^ and experimental^17,27,28^ results for single component BEC (*g*_12_ = 0).

#### *Case III*

: For a given inter-atomic repulsive interaction, or weak attractive interaction, i.e., *g* > −*c* and *g*_12_ > −*c*, the BEC is always stable, and the condition of phase transition can also been obtained analytically. From the stationary equation of Eq. (8), one can acquire $R={((g-{g}_{12}\mathrm{)/(2}{k}_{0}^{2}-{\rm{\Omega }}))}^{\mathrm{1/}D}\mathrm{/(2}\pi )$ for a crossover from *s* = 0 into *s* ≠ 0, i.e., from phase I into Phase II. By inserting it into the stationary equation of Eq. (11), the condition of phase transition is obtained as

15 $$\begin{array}{l}f={\omega }^{2}\,{[\frac{g-{g}_{12}}{{\mathrm{(2}\pi )}^{D\mathrm{/2}}\mathrm{(2}{k}_{0}^{2}-{\rm{\Omega }})}]}^{\mathrm{4/}D}-\frac{g+{g}_{12}}{\mathrm{2(2}\pi {)}^{D\mathrm{/2}}}\,{[\frac{g-{g}_{12}}{{\mathrm{(2}\pi )}^{D\mathrm{/2}}\mathrm{(2}{k}_{0}^{2}-{\rm{\Omega }})}]}^{\mathrm{(2}-D)/D}-1=0.\end{array}$$

**Figure 2:**
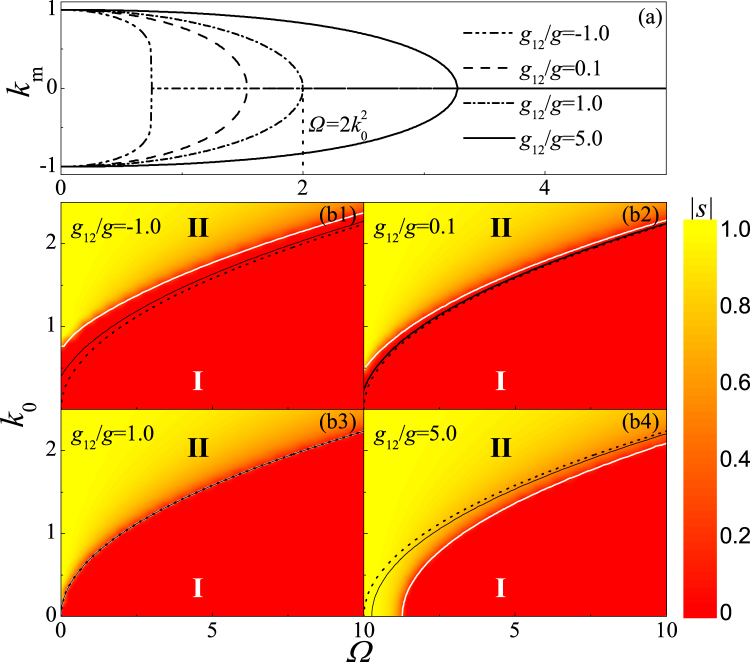
The phase diagram for 3D system. The first panel: *k*_m_ as a function of Ω for different interactions, where *k*_0_ = 1.0 and *g* = 10. The second panel and the third panel: the phase diagram in Ω − *k*_0_ plane for *g* = 10. The dot line shows ${\rm{\Omega }}=2{k}_{0}^{2}$, while the white and black solid line represents the phase transition boundary for 3D and 2D system, respectively.

Evidently, the phase structure of BEC can be elaborated by adjusting SOC, RC, interaction and the system’s geometric dimensionality. When *f* > 0, the system enters into phase I, on the contrary, the BEC is in phase II for *f* < 0, which is also clearly shown in Fig. 2. For a fixed *g*_12_/*g*, as the increase (decrease) of RC (SOC), the system transfers from phase II into phase I. When *g*_12_/*g* changes, the critical value ${({\rm{\Omega }}/{k}_{0}^{2})}_{{\rm{c}}}$ of phase transition from phase II to phase I is gradually changes, as well as ${({\rm{\Omega }}/{k}_{0}^{2})}_{{\rm{c}}} < 2$ for *g*_12_/*g* < 1 and ${({\rm{\Omega }}/{k}_{0}^{2})}_{{\rm{c}}}\mathrm{ > 2}$ for *g*_12_/*g* > 1. Moreover, the phase transition boundary of 2D system is closer to ${\rm{\Omega }}/{k}_{0}^{2}=2$ than that in 3D case, i.e., the phase transition is more sensitive to ${\rm{\Omega }}/{k}_{0}^{2}$ for a lower dimension system. Thus, the phase transition can more easily take place in 2D than 3D system.

**Figure 3:**
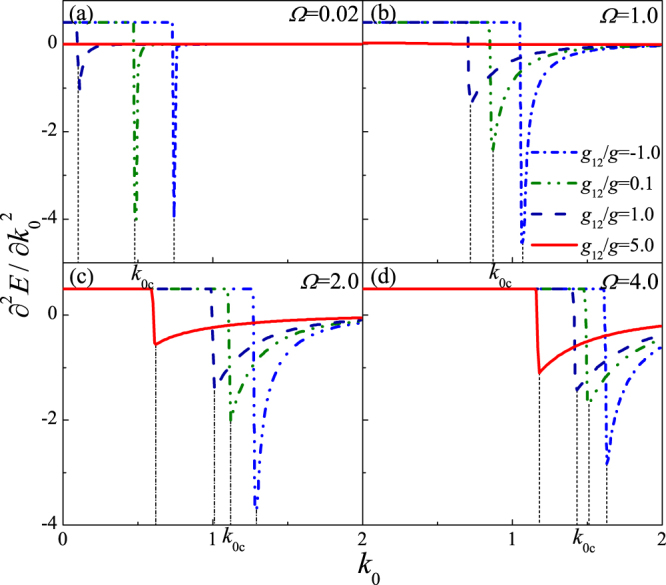
${\partial }^{2}E/\partial {k}_{0}^{2}$ as a function of *k*_0_ for *g* = 10 and *D* = 3.

To more clearly understand the nature of this phase transition, ${\partial }^{2}E/\partial {k}_{0}^{2}$ versus *k*_0_ is demonstrated in Fig. 3. The second derivative of energy has a jump at *k*_0_ = *k*_0*c*_, where the system exhibits a phase transition from phase I to phase II, and the jump point *k*_0*c*_ shifts right as the increase of *Ω* and shifts left as the increase of *g*_12_/*g*. These indicate that this phase transition has a second-order nature. The critical values *k*_0*c*_ are satisfied with the condition of phase transition *f* = 0 for interatomic repulsive interaction or weak attractive interaction, and also in agreement with Fig. 2.

#### *Case IV*

**Figure 4:**
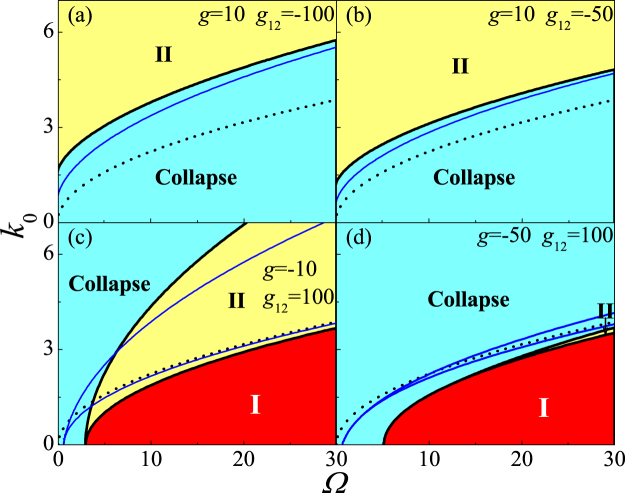
The stability diagram in Ω–*k*_0_ plane. The short dashed line corresponds to ${\rm{\Omega }}/{k}_{0}^{2}=2$. The blue and black solid line shows the border of stability diagram and phase transition of 2D and 3D system, respectively.

: For general case in 3D system, i.e., both repulsive and attractive interaction, although the ground state can not been obtained analytically, the complete phase diagram and stability diagram can be presented numerically by variational Eqs (7–11), which is explicitly demonstrated in *g* − *g*_12_ plane (Fig. 1) and Ω–*k*_0_ plane (Fig. 4). For strong inter-species attraction (Fig. 4(a,b)), there is ${\rm{\Omega }}/{k}_{0}^{2}={\eta }_{c1} < 2$ that the collapse occurs as ${\rm{\Omega }}/{k}_{0}^{2} > {\eta }_{c1}$, otherwise the stable phase II exists, where *η*_*c*1_ is determined by the coupling effects of the interatomic interaction, the harmonic trap potential and the system’s geometric dimensionality. In this case, the stabilizing mechanisms are illustrated that ${\rm{\Omega }}/{k}_{0}^{2} < {\eta }_{c1} < 2$ can lead to the BEC polarized, then stabilize the BEC with strong inter-species attractive interaction, and make the BEC enter into phase II. In other way, the weak RC and strong SOC (i.e., ${\rm{\Omega }}/{k}_{0}^{2} < {\eta }_{c1}$) can produce an effective inter-species repulsive interaction, which can balance the mean-filed inter-species attractive interaction to prevent the collapse and result in stable phase II. Further, *η*_*c*1_ in 3D system is smaller than that in 2D system, which indicates that the BEC is more unstable in 3D than 2D system. These are also demonstrated in Fig. 1(a2,b2,a3,b3), where the stability and phase boundaries are depicted in *g* − *g*_12_ plane. For strong intra-species attraction (Fig. 4(c,d)), the Ω–*k*_0_ plane is divided into three regions, the collapse region for ${\rm{\Omega }}/{k}_{0}^{2} < {\eta }_{c1}$, the stable phase II region for ${\eta }_{c1} < {\rm{\Omega }}/{k}_{0}^{2} < {\eta }_{c2}$, and stable phase I for ${\rm{\Omega }}/{k}_{0}^{2} > {\eta }_{c2} > 2$, where *η*_*c*2_ is defined by *f* = 0. In this case, ${\eta }_{c1} < {\rm{\Omega }}/{k}_{0}^{2} < {\eta }_{c2}$ can generate a spin polarized state, which can stabilize the collapse produced by strong intra-species attractive interaction, and make the BEC convert into phase II, whereas ${\rm{\Omega }}/{k}_{0}^{2} > {\eta }_{c2}$ can introduce a spin overlapped state, which can also stabilize the collapse produced by strong intra-species attractive interaction, and create a stable phase I. In other words, the strong RC and weak SOC (i.e., ${\rm{\Omega }}/{k}_{0}^{2} > {\eta }_{c1}$) produces an effective intra-species repulsive interaction, which can balance the mean-field intra-species attractive interaction then prevent the collapse. Compared with the 3D system, in 2D system, the region of collapse (stable BEC) decreases (increases), and the stability boundary is closer to ${\rm{\Omega }}/{k}_{0}^{2}=2$, namely, the BEC is more stable in 2D system. These are also evidently depicted in *g*–*g*_12_ plane (see Fig. 1(a1,b1,a3,b3)). In brief, the strong attractive collapse may be stabilized by elaborating the SOC, RC, harmonic trap potential, geometric dimensionality, and atomic interaction. Moreover, the collapse can be more easily stabilized in 2D than 3D system for all cases.

### Collapse dynamics

**Figure 5:**
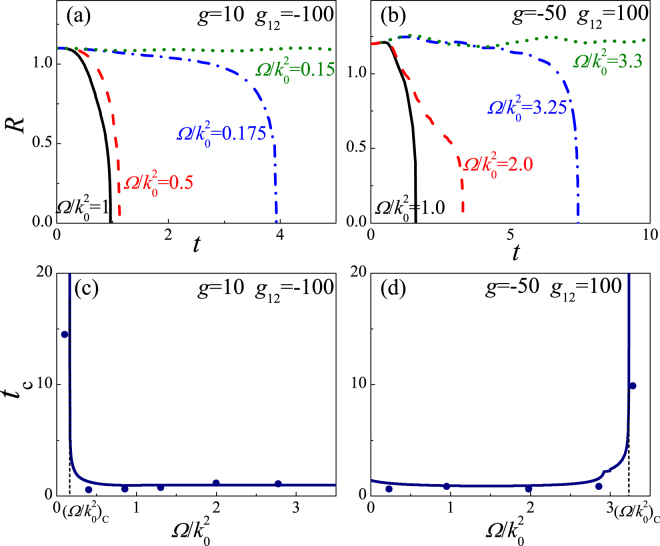
The collapse dynamics for 3D binary BEC with *k*_0_ = 2.0. (**a**,**b**) The time evolution of the BEC width for different ${\rm{\Omega }}/{k}_{0}^{2}$ and distinct phases. (**c**,**d**) The collapse time versus ${\rm{\Omega }}/{k}_{0}^{2}$ for distinct phases by means of the variational methods (the line) and the direct numerical simulation of Eq. (1) (the dots).

**Figure 6:**
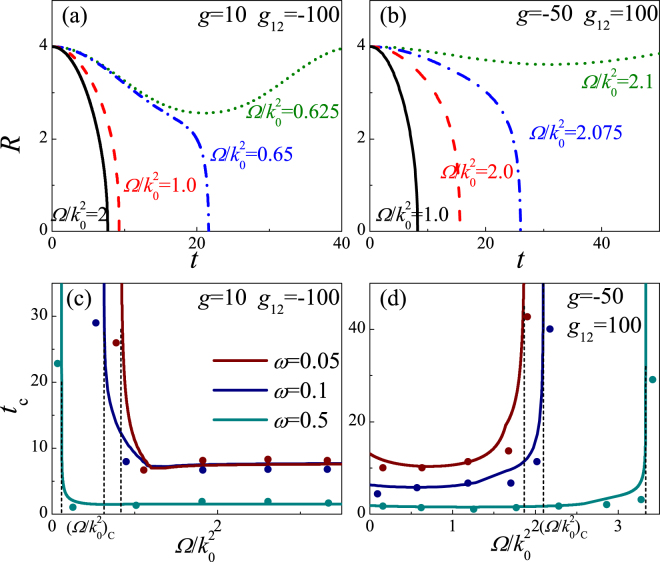
The collapse dynamics for 2D binary BEC with *k*_0_ = 2.0. (**a**,**b**) The time evolution of the BEC width for different ${\rm{\Omega }}/{k}_{0}^{2}$ and distinct phases. (**c**,**d**) The collapse time versus ${\rm{\Omega }}/{k}_{0}^{2}$ for distinct phases by means of the variational methods (the line) and the direct numerical simulation of Eq. (1) (the dots).

The breathing behavior of the BEC can be obtained by Eqs (7–9). Obviously, the breathing behavior is closely related to SOC, RC, and interatomic interaction. While the BEC width declines to zero in a limited time, the collapse occurs and the BEC is unstable, otherwise, the BEC is stable, and it is in phase I or phase II, which is demonstrated in Figs 5 and 6. Figure 5 depicts the collapse dynamics for 3D binary BEC. For strong inter-species attraction (Fig. 5(a,c)), when ${\rm{\Omega }}/{k}_{0}^{2} > {({\rm{\Omega }}/{k}_{0}^{2})}_{c}$, the BEC width declines to zero, and the collapse time has a finite value, thus the BEC is unstable and the collapse takes place; when ${\rm{\Omega }}/{k}_{0}^{2} < {({\rm{\Omega }}/{k}_{0}^{2})}_{c}$, the BEC width always oscillates around the equilibrium state, and the collapse time tends to infinity, thus the BEC is stable, meanwhile the system enters phase II. Namely, when ${\rm{\Omega }}/{k}_{0}^{2}$ is gradually close to ${({\rm{\Omega }}/{k}_{0}^{2})}_{c}$, the collapse time increases, and it tends to infinity for ${\rm{\Omega }}/{k}_{0}^{2} < {({\rm{\Omega }}/{k}_{0}^{2})}_{c}$, which indicates that the collapse speed is reduced as the decrease of ${\rm{\Omega }}/{k}_{0}^{2}$, and it slows to zero for ${\rm{\Omega }}/{k}_{0}^{2} < {({\rm{\Omega }}/{k}_{0}^{2})}_{c}$. For strong intra-species attraction (Fig. 5(b,d)), there is a critical value ${({\rm{\Omega }}/{k}_{0}^{2})}_{c}$ that the collapse occurs for ${\rm{\Omega }}/{k}_{0}^{2} < {({\rm{\Omega }}/{k}_{0}^{2})}_{c}$, while for ${\rm{\Omega }}/{k}_{0}^{2} > {({\rm{\Omega }}/{k}_{0}^{2})}_{c}$ the BEC is stable and it enters phase I or phase II, which depends on ${\rm{\Omega }}/{k}_{0}^{2}$ and *g*_12_/*g*. That is, the collapse speed decreases as the increase of ${\rm{\Omega }}/{k}_{0}^{2}$, and it droops to zero for ${\rm{\Omega }}/{k}_{0}^{2} > {({\rm{\Omega }}/{k}_{0}^{2})}_{c}$. Thus, the collapse time and the collapse speed can be manipulated by ${\rm{\Omega }}/{k}_{0}^{2}$, meanwhile the collapse produced by enough strong inter-species attractive interaction is stabilized by weak ${\rm{\Omega }}/{k}_{0}^{2}$, and large ${\rm{\Omega }}/{k}_{0}^{2}$ can prevent the collapse produced by enough strong intra-species attractive interaction.

The collapse dynamics for 2D case is demonstrated in Fig. 6, which is similar to 3D case. However, as the increase of the trapping frequency of transverse plane, i.e., the increase of *ω*, the stabilized collapse takes place at the smaller ${\rm{\Omega }}/{k}_{0}^{2}$ for strong inter-species attraction (see Fig. 6(c)) and the larger ${\rm{\Omega }}/{k}_{0}^{2}$ for strong intra-species attraction (see Fig. 6(d)). In the other hand, under the same conditions, as the increase of *ω* (which tends to 3D system step by step), the collapse time is gradually shorten, which leads to the larger collapse speed, i.e., the collapse more easily take places. In short, from 2D to 3D system, the increased collapse speed results in stabilizing the collapse produced by enough strong attractive interaction more difficultly.

**Figure 7:**
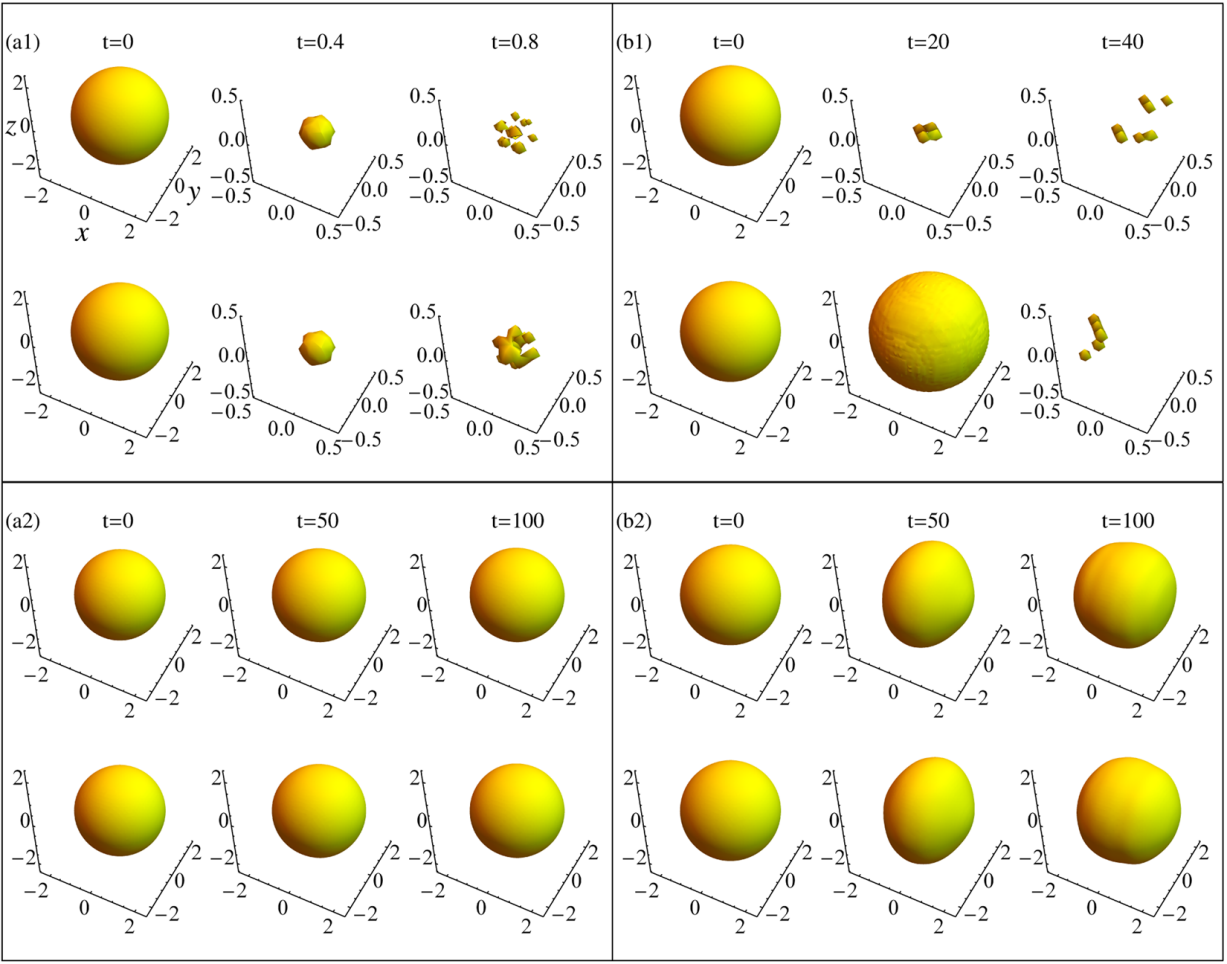
The evolution for the density profiles of 3D wave packets. |*ψ*_↑_| and |*ψ*_↓_| are plotted in the upper and lower panel in each subgraph, respectively. (**a1**): *g* = 10, *g*_12_ = −100 and *k*_0_ = Ω = 0. (**a2**): *g* = 10, *g*_12_ = −100, *k*_0_ = 2.0 and Ω = 0.5. (**b1**): *g* = −50, *g*_12_ = 100 and *k*_0_ = Ω = 0. (**b2**): *g* = −50, *g*_12_ = 100, *k*_0_ = 2.0 and Ω = 15.0.

The stability boundary and collapse time can also been obtained by the numerical simulation of GP equation, which is respectively demonstrated in Figs 1, 5 and 6 with dots. Moreover, Fig. 7 depicts the density profiles of 3D wave packet by the direct numerical dynamics evolution of Eq. (1) in order to conform the collapse dynamics. In absence of the SOC and RC, the binary BEC simultaneously quickly collapse due to strong inter-species attraction (see Fig. 7(a1)), which can be stabilized by a spin polarized state generated by ${\rm{\Omega }}/{k}_{0}^{2} < 2$, i.e., strong SOC and weak RC, while the BEC enters phase II (see Fig. 7(a2)). For strong intra-species, as without the SOC and RC, the more spin species collapses first, and later the less spin species collapses (see Fig. 7(b1)). The spin overlapping state induced by the strong RC and weak SOC (i.e., ${\rm{\Omega }}/{k}_{0}^{2} > 2$) can stabilize this collapse, and converts the BEC into phase I (see Fig. 7(b2)). The simulated results indicate that the variational predictions are appropriate.

**Figure 8:**
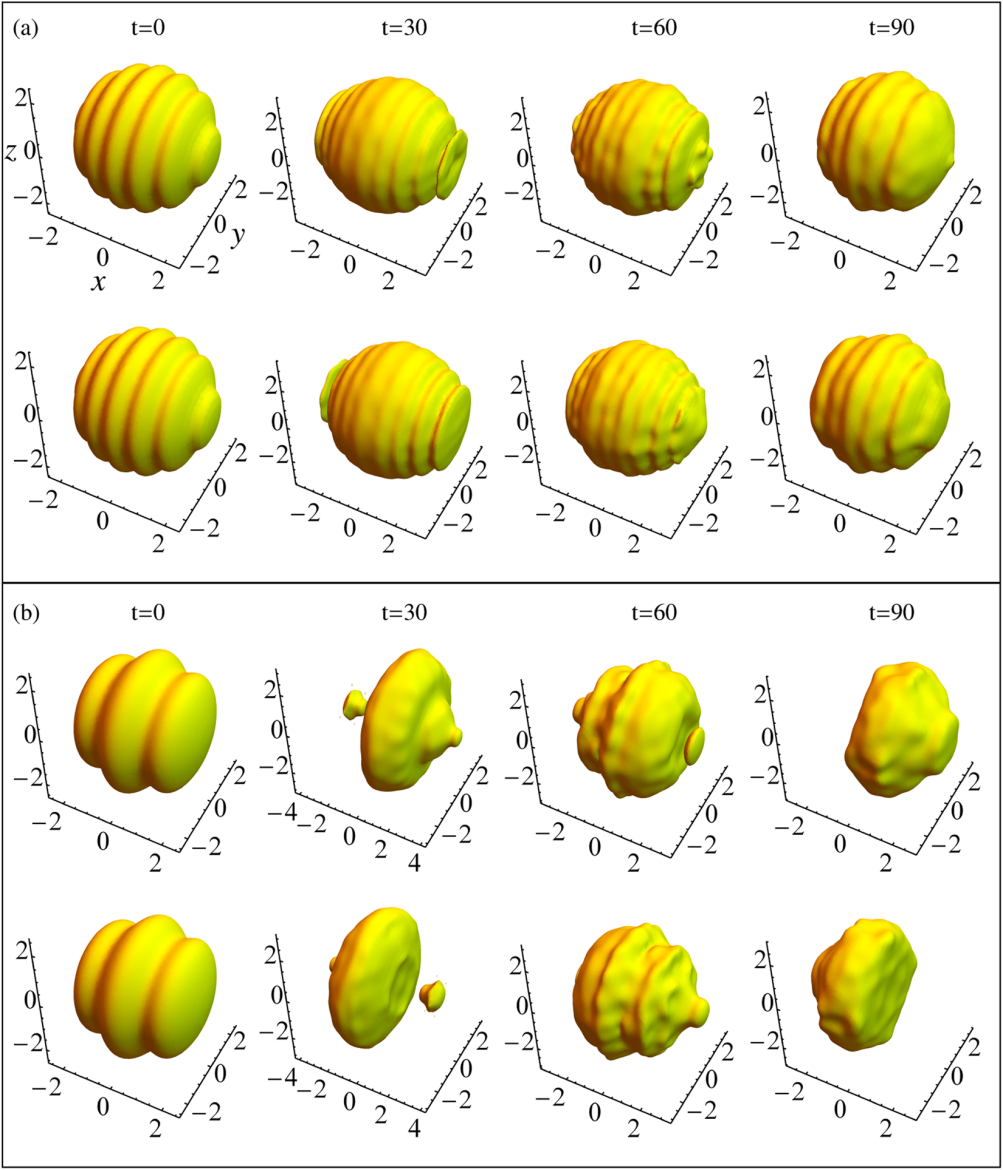
The evolution for the density profiles of 3D wave packets in the regions of the stripe phase (**a**) with Ω = 1.0, *k*_0_ = 2.0, *g* = 100, and *g*_12_ = 150, as well as the none stripe phase (**b**) with Ω = 1.0, *k*_0_ = 4.0, *g* = 100, and *g*_12_ = 80, respectively. |*ψ*_↑_| and |*ψ*_↓_| are plotted in the upper and lower panel in each subgraph, respectively.

However, due to the interference of the two species under the competitive effects between SOC and interatomic interaction, a new unique quantum phase, i.e., the stripe phase, has been predicted theoretically^12–15^ and observed experimentally^6^, which display a periodic density modulation spatially. The region for existing the stripe phase can be qualitatively obtained by similar variational methods where the trial wave function can be assumed as a superposition of two plane wave with momentum ±*k*_*x*_ corresponding to Eq. (3), i.e., the stripe phase only exists in the region of *g*_12_ < *g* and ${\rm{\Omega }}/{k}_{0}^{2}\ll 2$. As shown in Fig. 8, the dynamic evolutions for the density profiles of 3D wave packets in different phase regions are demonstrated. In the parameter region of the stripe phase (see Fig. 8(a)), the evolutions of the density profiles demonstrates as the spatially periodic density modulation along *x* direction because the SOC is only added in *x* direction, i.e., there indeed is a stable stripe phase in this region. Otherwise, there is not a stable stripe phase out of this region (see Fig. 8(b)). Thus, the occurrence of the stripe phase can modify the phase diagram of zero-momentum and plane wave phases, whereas their stability criteria can not be changed.

## Discussion

In conclusion, the combination of analytical and numerical methods depicts the phase and stability diagrams, while illustrates the stability mechanism of the trapped *D*-dimensional binary BEC with SOC. The dependence of phase transition and the stability of the system on SOC, RC, interatomic interaction, geometric dimensionality and external trap potential is presented in full parameter space. It is shown that the SOC-induced modification of dispersion relations can generate a spin polarized state or an overlapped state, which can compensate for the strong interatomic attraction, and stabilize the collapse, then changes the stability criteria. In particular, the condition of phase transition and stability criteria are gained, which represents that the phase transition and stability not only rely on interatomic interaction, SOC and RC, but also depend on the system’s geometric dimensionality. A full and deep understanding of the dependence of phase transition and stability mechanism on geometric dimensionality and external trap potential is demonstrated. It is also conformed by the collapse dynamics, which indicates that from 2D to 3D system, the mean-field attraction for inducing the collapse is reduced and the collapse speed is enhanced, then the collapse can be more easily stabilized in 2D system. That is, the collapse dynamics may be elaborated experimentally by adjusting the SOC, RC, geometric dimensionality, harmonic trap and atomic interaction.

## Methods

Here we have investigated the ground state properties of spin-orbit coupled Bose-Einstein condensate in a harmonic trap potential by using variational approach from the mean field Gross-Pitaevskii equations. The stability phase diagram and stability mechanism are analytically obtained, while collapse dynamics, collapse time and collapse speed are also presented, which both confirmed by the direct numerical simulations of Gross-Pitaevskii equations by means of the fourth-order Runge-Kutta scheme.
